# A protocol for a scoping review of language and cultural considerations when screening, assessing, and caring for people living with dementia or mild cognitive impairment from minoritised ethnic groups

**DOI:** 10.1371/journal.pone.0359314

**Published:** 2026-10-09

**Authors:** Leila Sharda, Kate Stalker, Deborah Buck, Olayemi Sadiku, Indu Surendran, Sanjana Buravalla, Emily Egan, Nomaan Zubair, Catherine Robinson, Ryan Cowley-Sharp, Charlotte Lennox, Jennifer Shaw, Stuart Ware, Lee Mulligan, Adam O’Neill, Louise Robinson, Katrina Forsyth

**Affiliations:** 1 Lancashire and South Cumbria NHS Foundation Trust, Preston, United Kingdom; 2 Division of Nursing, Midwifery, and Social Work, University of Manchester, Manchester, United Kingdom; 3 Pennine Care NHS Foundation Trust, Ashton-under-Lyne, United Kingdom; 4 Independent Lived Experience Consultant, United Kingdom; 5 Division of Psychology and Mental Health, University of Manchester, Manchester, United Kingdom; PLoS ONE, UNITED STATES OF AMERICA

## Abstract

The impact of language and culture on screening, assessment, and care for people from minoritised ethnic groups living with mild cognitive impairment (MCI) or dementia has previously been underestimated worldwide. A scoping review, informed by JBI guidelines and following the Preferred Reporting Items for Systematic Reviews and Meta-Analyses Protocol (PRISMA-P) checklist, will be conducted to determine what is known about the language and cultural considerations related to screening, assessing, and caring for people living with MCI/dementia from minoritised ethnic groups. Records will be obtained from Applied Social Sciences Index & Abstracts, Cumulative Index to Nursing and Allied Health Literature, Medical Literature Analysis and Retrieval System, APA PsycINFO, Social Sciences Full Text and Web of Science, and screened by two reviewers at title/abstract and full text for inclusion. The review will provide multi-agency practitioners, healthcare professionals, commissioners and policymakers with new insights related to the impact of language and culture when screening, assessing, and caring for people living with MCI/dementia from minoritised ethnic groups. The findings will contribute to current and future research ensuring people from minoritised ethnic groups receive equivalent health and social care for MCI/dementia.

**Scoping review registration:**

INPLASY202530018.

## Introduction

Dementia is highly prevalent worldwide, with 57 million people recorded as having dementia in 2021 and 10 million new diagnoses reported every year [1]. In actuality, dementia prevalence is likely to be much higher. Most studies are conducted in high-income countries and data from low-and middle-income countries (LMICs) is sparse [2,3].

People experiencing impairment in one or more cognitive domains without significant implications for activities of daily living may be diagnosed with mild cognitive impairment (MCI) [4]. While not all people experiencing MCI transition to dementia, MCI is a significant risk factor [5]. Dementia is diagnosed when there is ‘marked impairment in two or more cognitive domains relative to that expected’ [6], impacting on activities of daily living [6].

MCI and dementia may be prodromal for more than a decade [1]. Early diagnosis offers individuals sufficient time to make decisions about care and treatment, enabling plans for the future [2]. Therefore, from a societal perspective, early intervention may reduce health and social care costs by preventing unnecessary hospital admissions [1]. Given the total cost of dementia care in the United Kingdom (UK) is estimated to be 42 billion per year [3], dementia is a public health priority [1,7].

Current health and social care strategies to address dementia often overlook the specific needs of minoritised ethnic groups, offering generic policy recommendations instead of engaging with different community groups to fully understand cultural acceptability [8]. Although there is no internationally accepted definition of a minoritised group in law, minoritised ethnic groups can be considered the non-dominant population in each country in relation to ethnic, linguistic or religious characteristics [9]. Umbrella terms such as minority ethnic, ethnic minority and minoritised ethnic group should be used with caution [10].

When it is contextually justified to explore ethnicity as a broad category, in the UK the term ‘minoritised ethnic group’ is considered preferrable, in recognition that individuals are ‘minoritised through social processes of power and domination’ [10], also acknowledging that every person belongs to an ethnic group and ethnic minorities in one country are majorities elsewhere [10]. We use the term ‘minoritised ethnic group’ in this review; however, it is recognised that there is no universal approach and that healthcare systems need to respond to different minoritised people in a range of situations to provide quality intercultural care and support [11].

Data from healthcare records indicates some people from minoritised ethnic groups in the UK and the United States of America (USA) are utilising dementia services less frequently than the majority groups [1]. The incidence and prevalence of dementia are higher in some minoritised ethnic groups [1] e.g., in UK Black communities [12]. South Asian and Black people with dementia in the UK die younger than other ethnic groups [1]. There are serious and urgent concerns about the exclusion of minoritised ethnic groups from dementia care and a need to overcome the barriers currently inhibiting diagnosis [2,13].

While cognitive decline is not an inevitable part of biological aging [14], in some minoritised ethnic groups cognitive decline is normalised and there is little concept of ‘healthy aging’ e.g., among some Chinese Americans [15]. Cultural beliefs and perceptions about dementia and its aetiology may mean dementia is not recognised by some minoritised families [2]. Moreover, accessing help outside the family may be taboo and stigmatising [16] with a heavy burden placed on families, particularly women and children, to provide care at home [17].

When people do seek help, screening and assessment may be susceptible to cultural biases [18]. Indeed, most neuropsychological tests have been developed for Western White populations [19]. Screening tools such as the Rowland Universal Dementia Scale (RUDAS) designed to minimise the impact of cultural and language differences may still not offer a one size fits all approach [20] and the time required to administer the tool may deter use [21]. Many clinicians lack confidence overall to respond to the needs of minoritised groups [22], and as such, there may be deficiencies in assessments with bilingual and multilingual people [19].

People from minoritised ethnic groups may be at high risk of dementia being missed and consequently may experience delayed dementia treatment and care [2,19]. There is an urgent need to collate and synthesise [23] the international literature to better understand the language and cultural considerations related to screening, assessing, and caring for people who are living with dementia and MCI from different minoritised ethnic groups.

### Study purpose

The aim of this scoping review is to explore the impact of language and culture when screening, assessing, and caring for people living with dementia or MCI from minoritised ethnic groups and we will address the following research question:

What is the nature, range and extent of the literature regarding the impact of culture and language when screening, assessing or caring for people living with MCI or dementia?

## Materials and methods

### Study design

A scoping review informed by the JBI best practice guidance for the development of scoping review protocols [23] will be conducted by a multidisciplinary UK team and submitted for publication in an academic journal. This review protocol has been reported as per the Preferred Reporting Items for Systematic Reviews and Meta-Analyses Protocol (PRISMA-P) checklist [24] (S1 File). Items not applicable to scoping reviews are omitted.

### Eligibility criteria

Eligibility will be determined according to inclusion and exclusion criteria, established using the population, concept, and context (PCC) framework recommended by JBI [23]. PCC offers a useful framework to identify the key facets of scoping reviews, facilitating coherence between the review question and eligibility criteria [23]. The scope of this review is people of all ages living with dementia or MCI and/or informal and formal caregivers, of all genders and ethnicities (*population),* and language and cultural considerations in relation to screening, assessment and care *(concept)* in any setting globally *(context)*.

A pragmatic decision has been made to include only articles in the English Language and published from 2014. Key phenomena of interest will include considerations, challenges and adaptations to screening tools, interventions and services for people living with MCI or dementia, who are from minoritised ethnic groups. The review will consider articles focused on dementia knowledge as a phenomenon of interest when study participants have lived or caregiving experience of MCI or dementia. The Care Act (2014) in England mandates how social care and support is provided and although the scope of this review is international, we will also include articles related to maintaining social relationships as this constitutes care and support under this Act [25].

The review will include all primary research excluding case reports, conference proceedings and letters. Reviews will be excluded. Additionally, articles exploring acquired brain injury from birth, bilingualism, multilingualism, psychometric properties, translation of tools, racial differences between groups in the absence of cross-cultural considerations, access to health insurance, epidemiology, knowledge among the general public, and pre-clinical studies such as genetic, protein, and biomarker testing will be excluded as out of scope.

These criteria were developed iteratively via group consensus. Despite the importance of formal and informal caregiver experiences of supporting people living with dementia and MCI, these will be out of scope. The exception to this will be studies in which the data focuses specifically on caregivers’ beliefs or knowledge about screening, assessment or care of the individual they are supporting.

### Search strategy

The search strategy has been developed by the review team in collaboration with a University of *(redacted)* Library Information Specialist. Six bibliographic databases (Applied Social Sciences Index & Abstracts, Cumulative Index to Nursing and Allied Health Literature, Medical Literature Analysis and Retrieval System, APA PsycINFO, Social Sciences Full Text and Web of Science) were searched from January 2014 to February 2026 using adaptations of the following search string (S2 File):

Dementia or cognitive decline or alzheimer’s disease or mild cognitive impairment or MCI AND native tongue or mother tongue or dialect or diverse linguistic communities or language or culture or custom or heritage or tradition or cultural factor or culture minorities or cultural group or minority health or cultural-specific tools or immigrant group or ancestry or religion or ethnicity or nationality or nationalities or race or ethnic minorities or ethnic aspect or racial aspect or ethnic difference or race difference or racial minority or ethnic group or minority population or ethnically diverse communities or ethnic specific services or diverse ethnic minority communities or cultural adaptation* or cultural sensitivity or cultural identity or culturally linguistically diverse background* or minority ethnic communit* or ethnoracially diverse population* or culture-oriented assessment* or culturally tailor* or culturally diverse group* or indigenous health consideration* or culturally or linguistically informed healthcare or cultural competence AND caring or care or care planning or support or supporting or screening or screen or test or testing or detection or identification or assess or assessment or evaluation or diagnosis or diagnose or diagnosing.

### Data management

Following deduplication in Microsoft Excel (Version 16.95.3), all identified citations will be stored in a purposely designed Microsoft Excel spreadsheet (Version 16.95.3) to manage screening and selection.

### Study selection

Articles included following the title and abstract screen will be retrieved and screened against pre-determined inclusion criteria. The criteria will first be piloted by all reviewers and revised or clarified as necessary. After piloting, all texts will be read in full independently by two reviewers. Disagreements around eligibility will be resolved through team discussion. The search and selection results will be reported in full in the final report using a Preferred Reporting Items for Systematic Review and Meta-Analysis (PRISMA) flow diagram [26]. Screening is scheduled to be completed by 31^st^ October 2026.

### Data collection

A data extraction tool will be developed in Microsoft Excel (Version 16.95.3) and piloted on a subset of studies. Data will be extracted related to the study characteristics including publication type, study design, location, and setting, and sample characteristics such as age, ethnicity, study population, and experience of dementia, MCI or both. Additional data related to knowledge and beliefs about MCI and dementia, screening, assessment, and care including the use of tools and instruments as well as ethnic, language, and cultural considerations, perceived facilitators, challenges raised and adaptations to interventions and services will also be extracted. The data extraction will be undertaken by one reviewer and cross-checked by a second reviewer for accuracy and completeness. Data extraction is scheduled to be completed by 30^th^ November 2026.

### Data synthesis

The nature, range and extent of the literature will be tabulated and where possible, data items will be quantified and reported, e.g., publication type, study design, location and setting. The review team will then conduct a basic qualitative content analysis [27]. This will involve familiarisation with the data and a combination of inductive and deductive analysis [27]. We will then organise the data categorically creating narrative descriptive summaries depicting the impact of or considerations given to culture and language when screening, assessing or caring for people living with MCI and dementia.

## Discussion

Language and cultural considerations are highly relevant when screening, assessing, and caring for people from minoritised ethnic groups but can be overlooked as part of routine health and social care, resulting in disparities and health inequalities [28]. The mismatch between the incidence and prevalence of dementia reported in some minoritised ethnic groups in both the UK and the USA and the comparably low number of cases identifiable in clinical records represents an urgent and important concern [1].

We are currently unaware of any systematic scoping review of the international evidence related to language and cultural considerations when screening, assessing, and caring for minoritised ethnic groups living with dementia or MCI. This scoping review will address this knowledge and practice gap by systematically collating and synthesising the extant literature to examine what is known about the language and cultural considerations related to screening, assessing, and caring for people living with MCI or dementia from minoritised ethnic groups.

A potential limitation of this review is that it will involve a broad population, concept, and context requiring an extensive search, and encompassing a plethora of issues. However, this is an accepted feature of scoping reviews given their broader scope and research questions compared to, for example, systematic reviews [29]. A strength of this review is that the reviewers are from a range of disciplines including social work, psychology, nursing, medicine and psychiatry, and therefore represent ‘knowledge users’. The inclusion of knowledge users in scoping review teams has been considered to add focus, insight, and support prioritisation and the development of recommendations [30].

A further limitation of the review is that we will only be able to include publications in the English language due to resource limitations. We also had to make a pragmatic decision to exclude articles focused on the needs of caregivers. However, despite these limitations, our focus specifically on considerations impacting minoritised ethnic groups in each country of origin will enable this review to capture the breadth of understanding about the considerations, challenges raised and adaptations needed.

We will work with experts by experience to interpret our findings. We will also include articles concerning caregiver ‘knowledge’ of dementia or MCI to ensure that some aspect of the lived experience of caregiving is captured in this review. This is important because caregiver knowledge may impact the subsequent support a family member might receive.

This review is long overdue and will provide multi-agency practitioners, healthcare professionals, commissioners and policymakers with new insights related to the impact of language and culture when screening, assessing, and caring for people living with MCI or dementia from minoritised ethnic groups.

## Supporting information

S1 File Preferred Reporting Items for Systematic Reviews and Meta-Analyses Protocol (PRISMA-P) Checklist. (DOCX)

S2 File Search strategy. (DOCX)

## Abbreviations

LMICs: low- and middle-income countries
MCI: Mild Cognitive Impairment
PCC: Population, Concept, Context
PRISMA: Preferred Reporting Items for Systematic reviews and Meta-Analyses
RUDAS: Rowland Universal Dementia Scale
UK: United Kingdom
USA: United States of America
