# Supplementary material for: A protocol for a scoping review of language and cultural considerations when screening, assessing, and caring for people living with dementia or mild cognitive impairment from minoritised ethnic groups
Source: PLoS One. 2026 Oct 9;21(10):e0359314. doi: 10.1371/journal.pone.0359314 (PMC13652319; doi:10.1371/journal.pone.0359314)
Supplement: Supplementary file 2 [file pone.0359314.s002.docx]

**Additional File 2: Search Strategy**

*Note: all searches were by title or abstract*

(native tongue or mother tongue or dialect or diverse linguistic communities or language or culture or custom or heritage or tradition or cultural factor or cultural minorities or cultural group or minority health or cultural-specific tools or immigrant group or ancestry or religion or ethnicity or nationality or nationalities or race or ethnic minorities or ethnic aspect or racial aspect or ethnic difference or race difference or racial minority or ethnic group or minority population or ethnically diverse communities or ethnic specific services or diverse ethnic minority communities or cultural adaptation* or cultural sensitivity or cultural identity or culturally linguistically diverse background* or minority ethnic communit* or ethnoracially diverse population* or culture-oriented assessment* or culturally tailor* or culturally diverse group* or indigenous health consideration* or culturally and linguistically informed healthcare or cultural competence)

AND

(dementia or cognitive decline or alzheimer's or mild cognitive impairment or MCI)

AND

(caring or care or care planning or support or supporting or screening or screen or test or testing or detection or identification or assess or assessment or evaluation or diagnosis or diagnose or diagnosing)

**OVID (Medline and PsycINFO)**

(native tongue or mother tongue or dialect or dialects or diverse linguistic communit* or language or languages or culture or cultures or custom or customs or heritage or heritages or tradition or traditions or cultural factor* or cultural minorities or cultural group* or minority health or cultural-specific tool* or immigrant group* or ancestry or religion* or ethnicity or nationality or nationalities or race or ethnic minorit* or ethnic aspect* or racial aspect* or ethnic difference* or race difference* or racial minorit* or ethnic group* or minority population* or ethnically diverse communit* or ethnic specific service* or diverse ethnic minority communit* or cultural adaptation* or cultural sensitivity or cultural identit* or culturally linguistically diverse background* or minority ethnic communit* or ethnoracially diverse population* or culture-oriented assessment* or culturally tailor* or culturally diverse group* or indigenous health consideration* or “culturally and linguistically informed healthcare” or cultural competenc*)

AND

(dementia or cognitive decline or alzheimer's or mild cognitive impairment or MCI)

AND

(caring or care or care planning or support or supporting or screening or screen or test or testing or detection or identification or assess or assessment or evaluation or diagnosis or diagnose or diagnosing)

**EBSCOHOST (CINAHL and Social Sciences- Full Text), Web of Science, ASSIA**

(“native tongue” or “mother tongue” or dialect or dialects or “diverse linguistic communit*” or language* or culture* or custom or customs or heritage* or tradition or traditions or “cultural factor*” or “cultural minorit*” or “cultural group*” or “minority health” or “cultural-specific tool*” or “immigrant group*” or ancestry or religion* or ethnicity or nationalit* or race or “ethnic minorit*” or “ethnic aspect*” or “racial aspect*” or “ethnic difference*” or “race difference*” or “racial minorit*” or “ethnic group*” or “minority population*” or “ethnically diverse communit*” or “ethnic specific service*” or “diverse ethnic minority communit*” or “cultural adaptation*” or “cultural sensitivity” or “cultural identit*” or “culturally linguistically diverse background*” or “minority ethnic communit*” or “ethnoracially diverse population*” or “culture-oriented assessment*” or “culturally tailor*” or “culturally diverse group*” or “indigenous health consideration*” or “culturally and linguistically informed healthcare” or “cultural competenc*”)

AND

(dementia or cognitive decline or alzheimer's or mild cognitive impairment or MCI)

AND

(caring or care or care planning or support or supporting or screening or screen or test or testing or detection or identification or assess or assessment or evaluation or diagnosis or diagnose or diagnosing)
